# Endoscopic closure of plastic biliary stent-induced duodenal perforation opposite the major papilla using anchor pronged clips

**DOI:** 10.1055/a-2970-8269

**Published:** 2026-10-19

**Authors:** Daiki Hiraga, Takashi Abe, Takayuki Baba, Riku Yodo, Masanori Tokoro, Takayuki Nagai, Kazuhiro Mizukami

**Affiliations:** 1 Department of GastroenterologyOita Kouseiren Tsurumi HospitalBeppuJapan; 2 General Medicine and GastroenterologyFaculty of Medicine, Oita UniversityYufuOitaJapan

A 79-year-old woman presented with obstructive jaundice. Laboratory investigations demonstrated markedly elevated cholestatic parameters, including total bilirubin of 11.7 mg/dL (normal range: 0.4–1.5 mg/dL), aspartate aminotransferase of 120 U/L (13–30 U/L), alanine aminotransferase of 125 U/L (7–23 U/L), alkaline phosphatase of 831 U/L (38–113 U/L) and γ-glutamyl transpeptidase of 532 U/L (9–32 U/L). Contrast-enhanced abdominal computed tomography (CT) and magnetic resonance cholangiopancreatography revealed bile duct wall thickening just distal to the cystic duct confluence, with upstream biliary dilatation ( Figs. 1 and 2 ). Endoscopic retrograde cholangiopancreatography demonstrated a stricture extending from the distal bile duct to the common hepatic duct ( Fig. 3a ). Following endoscopic sphincterotomy, transpapillary biopsy revealed adenocarcinoma. Subsequently, a 7-Fr×7-cm straight plastic biliary stent and a 5-Fr×3-cm pancreatic stent were placed ( Fig. 3b and c ). Two days later, the patient developed abdominal pain with an elevated C-reactive protein level (15.3 mg/dL). CT revealed free air around the second portion of the duodenum ( Fig. 4 ). Emergency upper gastrointestinal endoscopy revealed a duodenal perforation opposite the major papilla caused by the tip of the biliary stent, therefore closure with Mantis clips (Boston Scientific, Marlborough, MA, USA) was attempted. The endoscope was exchanged for a duodenoscope, and both stents were removed because of interference from the perforating biliary stent. The straight biliary stent was replaced with a 7-Fr×7-cm double-pigtail plastic biliary stent to prevent contact with the mucosal surface. The endoscope was then exchanged again for a forward-viewing endoscope, and the perforation was successfully closed using Mantis clips. Duodenography confirmed the absence of contrast leakage ( Video 1) . The patient’s postoperative course was uneventful, and she was discharged on postoperative day 17.

**Fig. 1:**
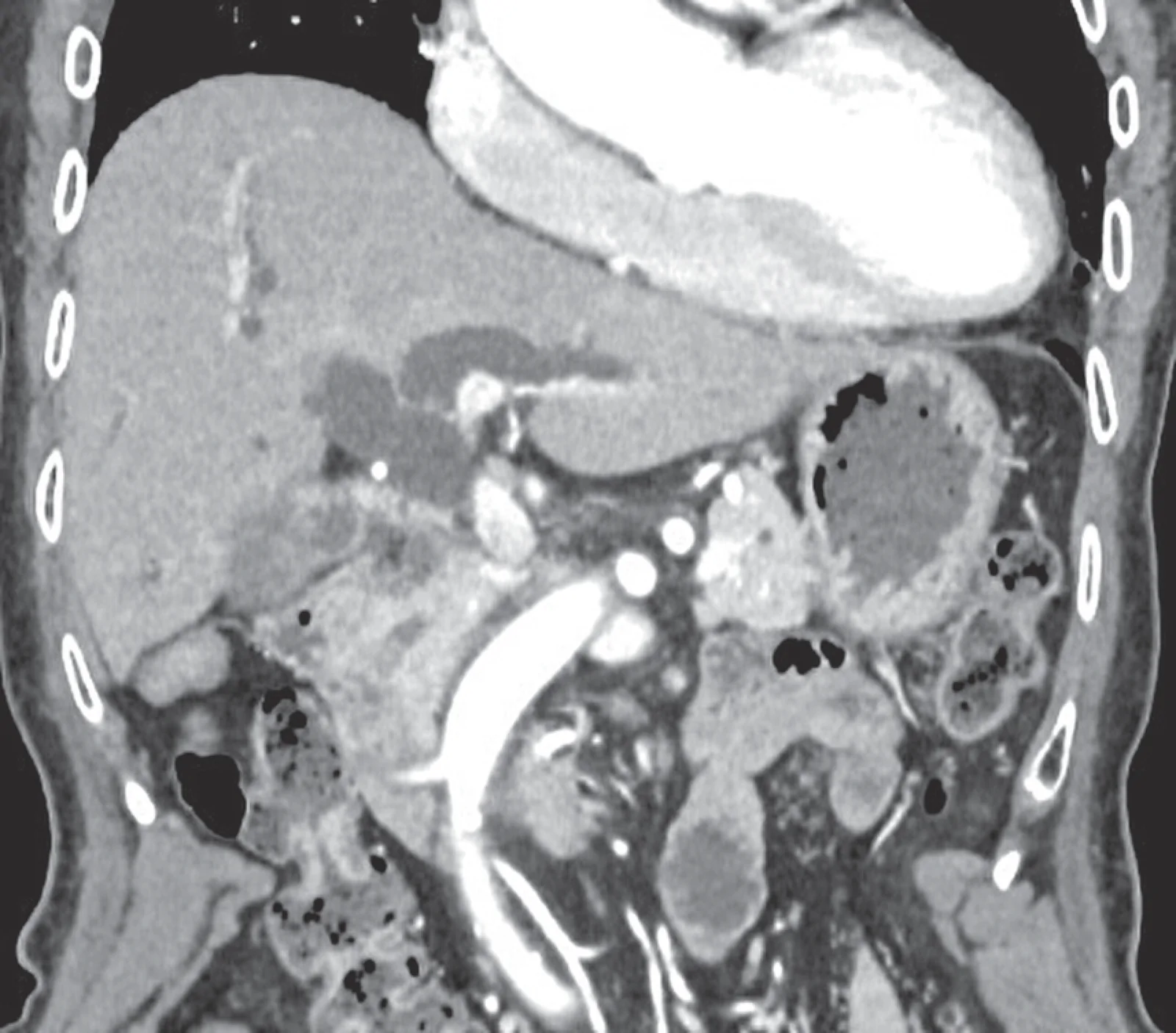
Reconstructed coronal contrast-enhanced abdominal Computed tomography image showing bile duct wall thickening just distal to the cystic duct confluence, with upstream biliary dilatation.

**Fig. 2:**
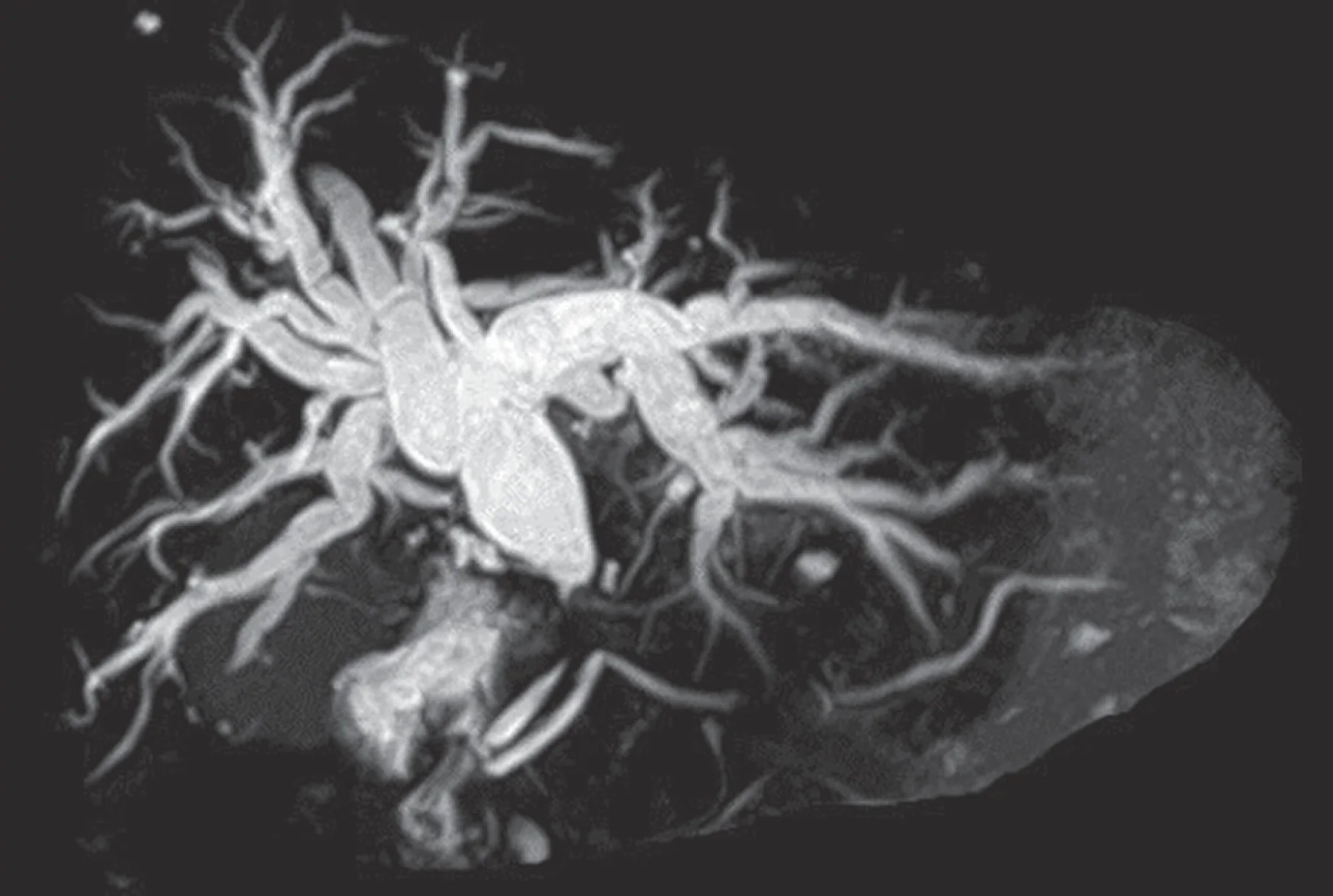
Magnetic resonance cholangiopancreatography image showing bile duct wall thickening just distal to the cystic duct confluence, with upstream biliary dilatation.

**Fig. 3:**
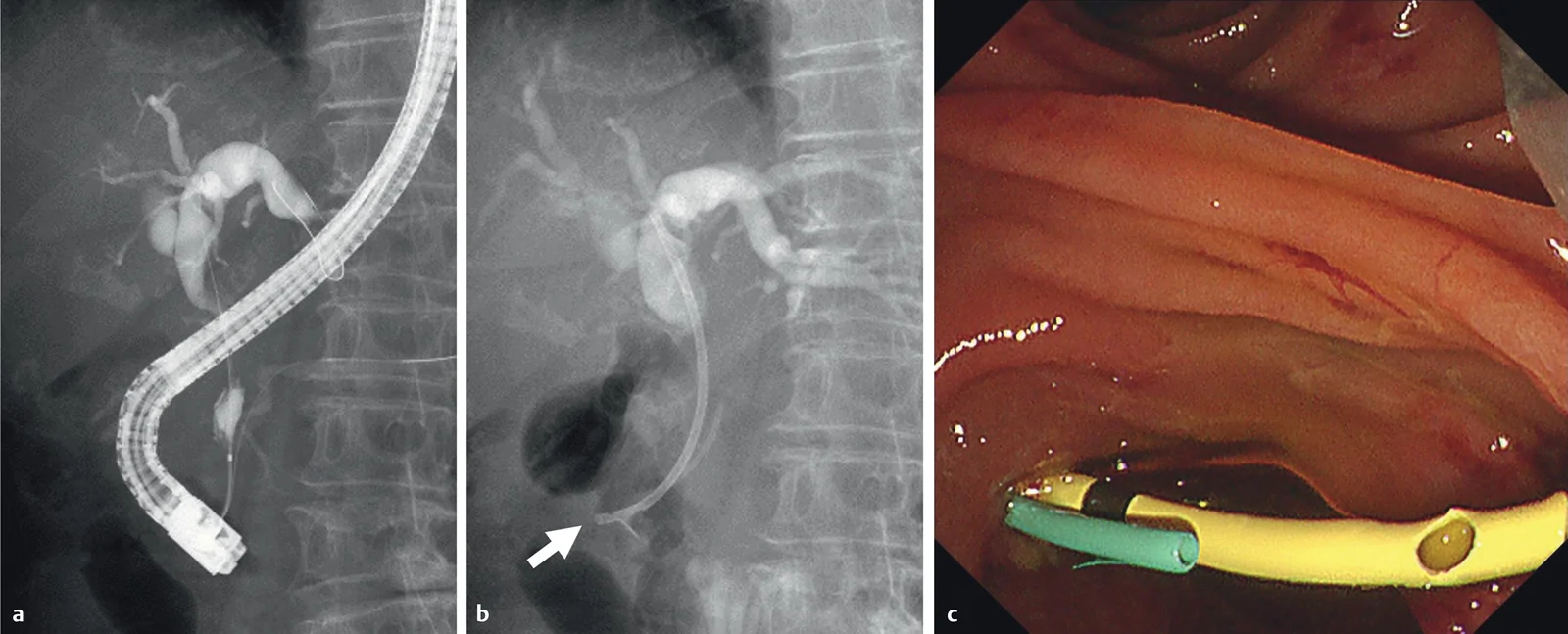
Endoscopic views. A guide wire has been inserted into the bile duct and pancreatic duct ( **a** , **b** ). Cholangiography demonstrated a stricture extending from the distal bile duct to the common hepatic duct ( **a** ). A 7-French×7-cm straight plastic biliary stent and a 5-French×3-cm pancreatic stent were placed ( **b** ). Endoscopic view at the end of the procedure **(c** ). The tip of the biliary stent is in contact with the duodenal wall opposite the major papilla (arrow).

**Fig. 4:**
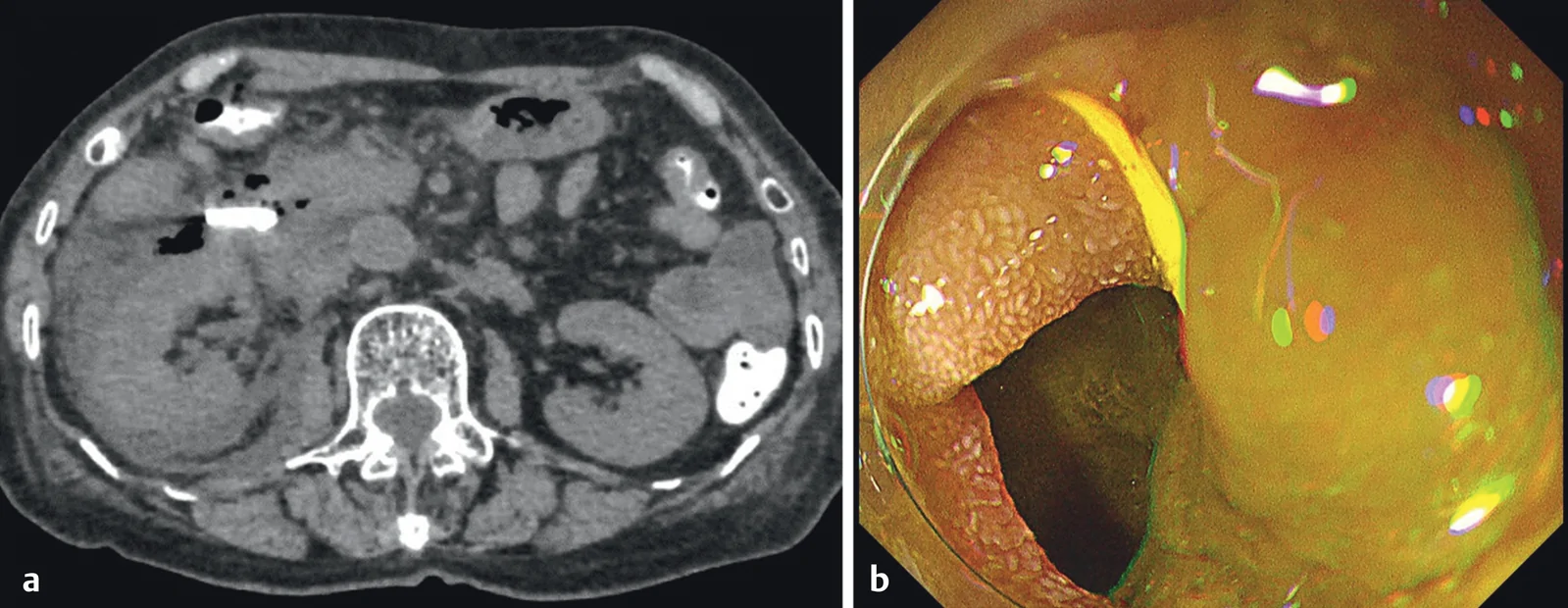
Abdominal computed tomography image showing free air around the descending duodenum (arrow), suggesting duodenal perforation caused by the biliary stent ( **a** ). Endoscopic image showing the duodenal perforation opposite the major papilla caused by the tip of the biliary stent ( **b** ).

**Video 1** Successful endoscopic closure of a plastic biliary stent-induced duodenal perforation opposite the major papilla using Mantis clips.


Biliary stent-induced duodenal perforation is extremely rare. If stent-related duodenal perforation occurs, endoscopic closure using Mantis clips may be an effective treatment option.

PM-Endoscopy_UCTN_Code_TTT_1AO_2AO

## Declaration of GenAI Use

The authors confirm that no Generative AI was used in the preparation of this manuscript.

